# Modeling and analysis of 100 kW two-stage three-phase grid-connected PV generation system under absurd atmospheric and grid disturbances

**DOI:** 10.1371/journal.pone.0323269

**Published:** 2025-06-20

**Authors:** Muhammad Hafeez Mohamed Hariri, Mohamed Salem, Mohamad Kamarol Mohd Jamil, Mohamad Nazir Abdullah, Mohd Khairunaz Mat Desa

**Affiliations:** 1 School of Electrical and Electronic Engineering, Universiti Sains Malaysia (USM), Nibong Tebal, Penang, Malaysia; Institute of Aviation Engineering and Technology, EGYPT

## Abstract

The sustainable growth of renewable energy sources, especially photovoltaic (PV) driven electricity generation, is expected to grow exponentially over the next few years. The extraction of grid parameters such as the line voltage’s magnitude, phase angle, and phase sequence, are crucial for the effective control of PV-grid synchronization. The existing grid synchronization technique such as a conventional phase-locked loop (PLL) is unable to provide an accurate fundamental frequency positive sequence under various types of grid fault conditions. Therefore, the main purpose of this article is to model and analyze the introduction of cascaded delay signal cancelation (CDSC) for a 100 kW two-stage three-phase grid-connected PV generation system under absurd atmospheric and various grid disturbances. The performance of the CDSC is benchmarked with a conventional structure of PLL as well as a double second-order generalized integrator (DSOGI). The modeling and analyses of these selected grid synchronizers were performed in a Matlab-Simulink (R2020b) environment.

## 1. Introduction

Renewable energy (RE) is defined as clean energy that comes from natural renewable resources, such as wind, hydro, biomass, geothermal, tidal, and solar (photovoltaic). The future energy pathways based on existing analysis show that better access to energy, clean air, and preserved energy security can be simultaneously achieved while mitigating harmful climate changes [1]. Amongst the mentioned RE, hydro and photovoltaic (PV) are among the most prominent ones due to the huge potential they offer [2,3]. The sustainable future of PV is elaborated in detail in [4,5]. Stand-alone and grid-connected PV (GPV) generation systems are the two primary categories of solar energy systems. Both systems’ implementations and objectives share a number of similarities and distinctions. A GPV system is a separate, decentralized power system that is linked to a transmission and distribution network for electricity. The integration of GPV systems to the utility grid keeps growing over the years. It requires a proper grid synchronization mechanism with a fast transient response and poses a high resilience against various grid disturbances to further improve the reliability and the quality of power transfer from the PV system to the utility grid [6,7].

There are two main circuit configurations of GPV systems, namely single-stage and two-stage systems. Both systems share similarities in their purposes and implementations but differ significantly. Based on the literature, single-stage three-phase GPV generation systems suffer from several drawbacks such as excessive installation costs, high complexity, and a greater input current stress on the series inductors [8]. In addition, the performance of the MPPT of the GPV system particularly on the tracking efficiency under various weather conditions is still being debated. For instance, the operating point of PV using the famous P&O techniques creates oscillations in the region of the maximum power point (MPP), giving rise to the waste of energy [9,10]. Furthermore, several technical problems regarding stability, safety, and power quality issues such as harmonics, frequency fluctuations, and others have cropped up owing to the increased penetration of GPV systems [11,12]. Several problems have arisen with regard to this issue due to the integration of large amounts of GPV systems at different locations (randomly installed) and possessing different voltage profiles which leads to voltage imbalance along the feeders [13–15]. At the moment, the voltage rise should not exceed the 2% limit as stated by the grid code standards [16,17]. In some cases, there are faulty conditions on the transmission line that cause the grid variables (voltage amplitude, phase angle, and frequency) to reach and exceed threshold limits, consequently causing the controllers to malfunction [18–20]. Moreover, the GPV systems which comprise a portion of power electronics devices could transfer the generated PV power without proper guidelines, causing a huge influx of total harmonic distortion (THD) at the point of common coupling (PCC) [21]. The effect of harmonic content on transmission lines is enormous as discussed in [22]. In a GPV system, the synchronization process should be fast, effective, and robust even with the presence of miscellaneous types of sources and load profiles along the feeder. Up-to-date, there are numerous synchronization methods proposed in the literature to synthesize grid information. Among these, the zero-crossing strategy (ZC) remains the simplest strategy even though it is not the most accurate strategy under non-ideal conditions [23]. During voltage variation or the presence of harmonics, the ZC point is detectable only every half period of the grid voltage or frequency. Consequently, due to insufficient detections, the dynamic performance of the controller will degrade [24]. Moreover, the existing PLL synchronization mechanisms of the GPV system face difficulties in providing the accurate value of grid information during fault conditions [25]. A combination of suitable and latest controllers combined with a two-stage three-phase inverter structure configuration could improve the efficiency and the reliability of the proposed system in the event of absurd atmospheric and various grid fault conditions. A comprehensive comparative analysis that highlights how this proposed research work differs from prior studies is summarized in Table 1.

**Table 1:** A comparative analysis of the proposed research work differs from prior studies.

Criteria	Proposed System	[26]	[27]	[28]
Title	Modeling and Analysis of 100 kW Two-Stage Three-phase Grid-Connected PV Generation System under Absurd Atmospheric and Grid Disturbances	Modeling and Analysis of Three‑phase Grid‑tied Photovoltaic Systems	A Control Strategy for a Three-Phase Grid Connected PV System under Grid Faults	Modeling and Simulation for an 8 kW Three-Phase Grid-Connected Photo-Voltaic Power System
Published Year	Est. 2025	2023	2019	2017
System Size	100kW	10kW	100kW	8kW
Type of PV System	Two-stage, three-phase grid-connected PV system	Two-stage, three-phase grid-connected PV system	Two-stage, three-phase grid-connected PV system	Single-stage, three-phase grid-connected PV system
Main Focus	Various Atmospherics profile and Grid Disturbances	Effect of Atmospherics profile on generated output power	Under Grid Faults	Steady-state conditions
Control Strategy	Cuckoo Search (CS) MPPT and CDSC	INC MPPT and PLL	INC MPPT and PLL	P&O MPPT and SPLL
Simulation or Experimental Approach	Simulation- based	Simulation-based	Simulation-based	Simulation-based
PV-Grid Connection Type	Grid-connected PV system	Grid-connected PV system	Grid-connected PV system	Grid-connected PV system
Key Contributions	Provides detailed model analysis and better solutions to mitigate atmospheric and grid disturbances’ effect on a large-scale two-stage three-phase grid-connected PV system.	A five-level VSI interconnected to a grid controlled by SRF structure with PD multicarrier modulation technique is analyzed and presented	The implemented control strategy provides a switch between MPPT mode and non-MPPT mode to ensure the protection of the power converters	PQ controller is presented and studied for PV-grid connection control.

Hence, a proposed 100 kW two-stage three-phase GPV generation system is presented in this article. The overall system response towards the transient conditions due to the presence of absurd atmospheric profiles and different types of faults such as voltage unbalance, voltage dip, total harmonic distortions, and line faults in grid power line becomes the main concern in this research in assessing the ability of the proposed system, as well as the overall performance of the designated local controllers to compensate the changes. The overall results show that the proposed 100 kW two-stage three-phase GPV generation system comprises controllers of the Cuckoo Search (CS) MPPT technique; the SVPWM switching topology in combination with the CDSC synchronization mechanism is the most effective PV-grid system configuration as it proved to have better MPPT tracking efficiency of 96 % and requires only 0.05 s to reach steady-state in the course of transient, ${THD}_{i}$ level of 2.06 %, and the most important is the proposed system works efficiently even in the case of grid fault conditions.

This article is structured as follows: Section 2 reveals the mathematical modeling of PV; Section 3 presents the MPPT; Section 4 describes the technical specifications of DC-DC boost converter; Section 5 covers three-phase VSI; Section 6 outlines the details of SVPWM; Section 7 presents the design parameters of interfacing line filters; Section 8 overviews grid synchronization mechanisms; Section 9 explains the CDSC technique; Section 10 summaries the system’s specifications; Section 11 covers results; Section 12 concludes the overall system performances.

## 2. Mathematical modeling of photovoltaic

**Fig 1:**
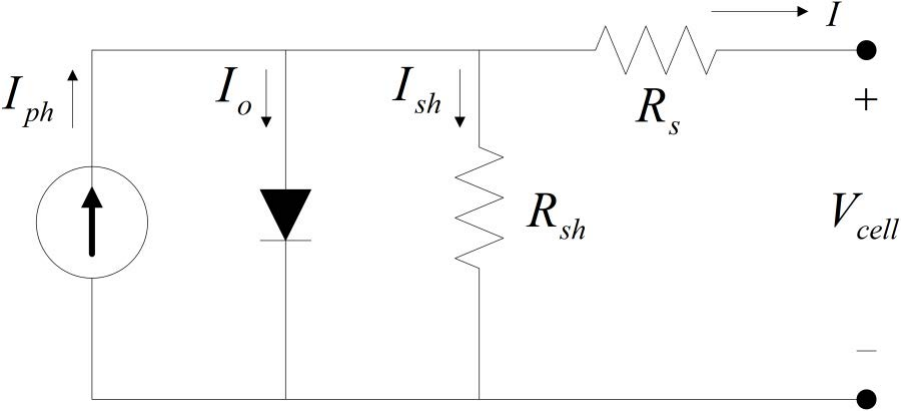
PV cell modeled as a diode circuit.

The PV cell circuit is generally depicted as a single-diode model that is made up of four major components – a photocurrent source $I_{ph}$, a series resistor, $R_{s},$ a diode that is parallel to the sources, and a shunt resistor, $R_{sh}$ (see Fig 1).

The current flow through a diode is defined as $I_{d}$, $I_{sh}$ is shunt current, the series resistance, $R_{s}\;$ is the internal resistance of the PV cell. Leakages at the junction lead to shunt resistance, $R_{sh\;,}\;$ and $V_{cell}$ is the cell voltage of the PV. The I−V  features of a PV cell are determined as follows:


$$\;I=I_{ph}-I_{d}-I_{sh\;\;}$$ (1)

$$I_{ph}=\frac{G}{1000}\left(I_{sc}+k_{T}(T-298)\right)$$ (2)

Here, $I_{ph}$ stands for photo-current, G is the solar irradiation in $W/m^{2}$, T stands for the operating temperature of the PV cell, $k_{T}$ is short-circuit current of cells at 25 °C and $1000\;W/m^{2}$.


$$I_{d}=I_{o}\left[\exp\left(q\frac{V_{cell}+R_{s}I}{kT}\right)-1\right]$$ (3)

Here, $I_{o}$ is the diode saturation current, q is electron charge with the value of $1.6\times 10^{-19}C$, and k is Boltzmann constant $1.3805\times 10^{-23}\;J/K.$


$$I_{o}=\;I_{rs}{\left(\frac{T}{T_{r}}\right)}^{3}\exp\left[\frac{qE_{g}}{Bk}\left(\frac{1}{T}-\frac{1}{T_{r}}\right)\right]$$ (4)

$$I_{rs}=\frac{I_{sc}}{\left[\exp\left(\frac{qV_{oc}}{BkT}\right)-1\right]}$$ (5)

$$I_{s}\;=AT^{3}\exp\left(\frac{-qE_{g}}{BkT}\right)$$ (6)

Here, $I_{rs}\;$ stands for reserve saturation current, $T_{r}$ is the nominal temperature of $298K,\;E_{g}\;$ is the bandgap energy of a semiconductor  1.1eV, A and B constants are ideality factors of the diode that vary between 1.0 and 1.6, $V_{oc}$ is an open-circuit voltage of the PV cell.

The operating voltage of PV cells can be improved by connecting them in series while the current capacity can be enhanced by connecting them in parallel. Hence, the calculation of the total current in the PV module is done as follows:


$$I=n_{p}I_{ph}-n_{p}I_{s}\left\lceil \exp\left(\frac{q\left(V_{cell}+R_{s}I\right)}{n_{s}BkT}\frac{n_{s}}{n_{p}}\right)-1\right\rceil -\frac{V_{cell}\frac{n_{p}}{n_{s}}+R_{s}I}{R_{sh}}$$ (7)

where $n_{s}$ represent the number of serially connected PV cells and $n_{p\;}$ are the number of PV cells connected in parallel. The simulation process in this work is done using the BP275F solar module as the reference PV model.

## 3. Maximum power point tracking

**Fig 2:**
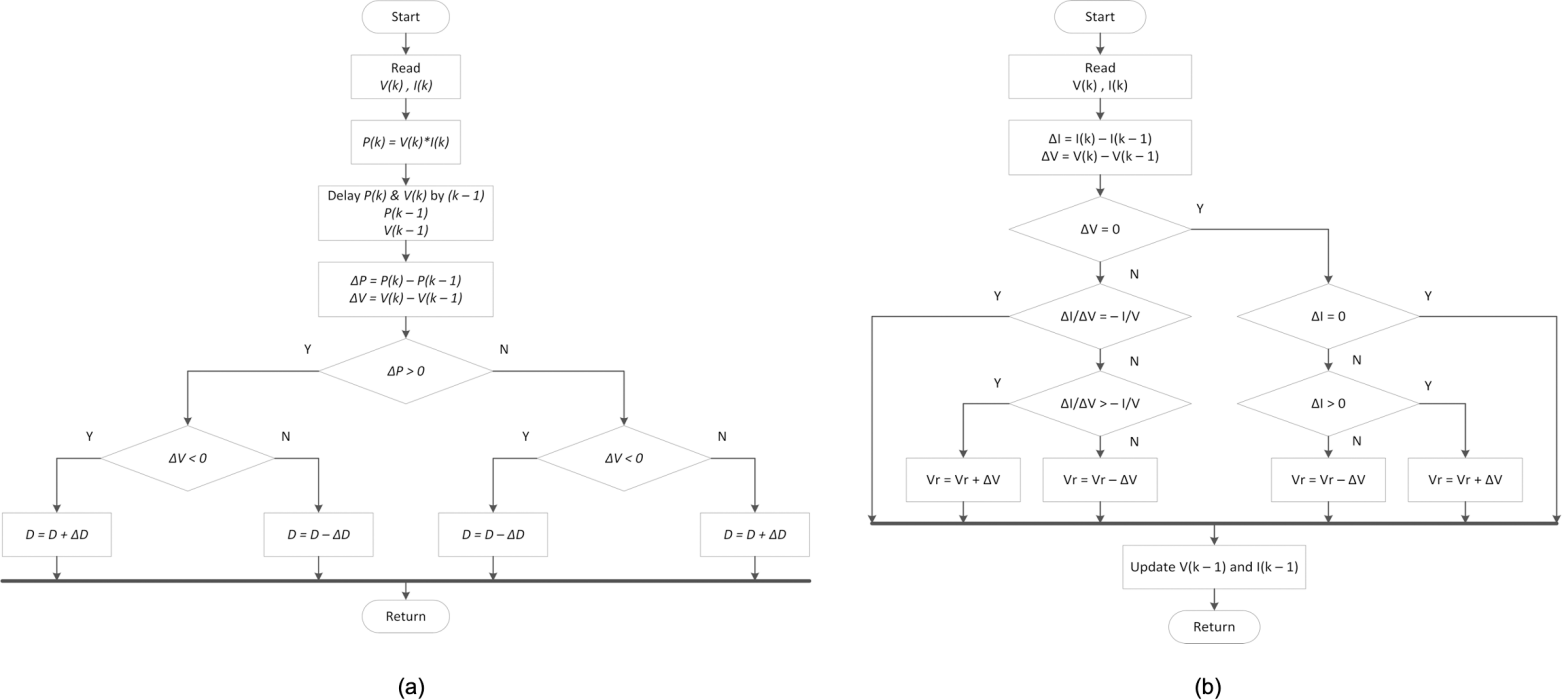
(a) The flowchart diagram of the P&O and (b) INC MPPT technique.

Two categories of MPPT techniques are implemented in this research work. A conventional approach is represented by perturbation and observation (P&O) and incremental conductance (INC) whereas the advanced computing methodology is represented by Cuckoo Search (CS). The P&O algorithm flowchart is illustrated in Fig 2(a). Meanwhile, the INC MPPT technique is founded on the notion that, at the MPP, the PV module power-voltage curve has a slope value of zero, a negative slope on the right of the MPP, and a positive slope value on the left of the MPP. The INC method is depicted in the flowchart diagram in Fig 2(b).

**Fig 3:**
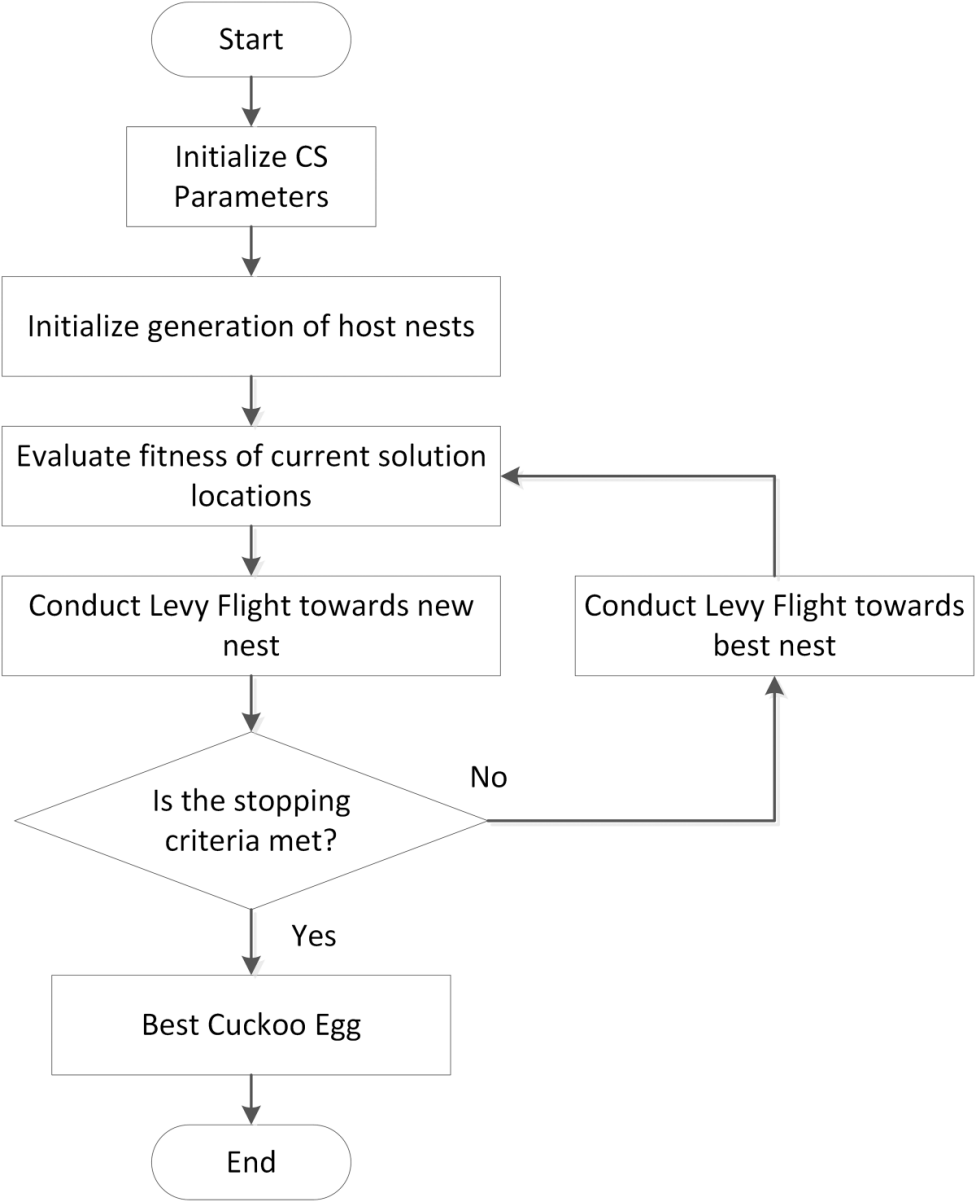
The flowchart diagram of the CS algorithm technique [30].

On the other hand, CS algorithm as illustrated in Fig 3 can be classified as one of the metaheuristic algorithms in which its working principle is based on the nature-inspired brood parasitism of some cuckoo bird species along with Levy flights random walks [29].

## 4. Technical specification of DC-DC boost converter

**Fig 4:**
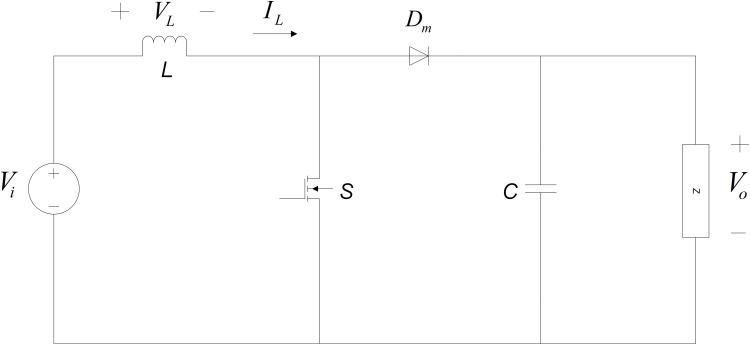
Circuit diagram of the DC-DC boost converter.

The DC-DC boost converter is a preferable DC-DC converter topology as it provides a higher voltage level on the output side which is later been projected as a DC input to the three-phase VSI inverter. The typical circuit diagram of the DC-DC boost converter is illustrated in Fig 4.

The mathematical expression of output voltage, $V_{o}$ in terms of input voltage, $V_{i}$ is as follows:


$$V_{o}=\;\frac{V_{i}}{1-D}\;\;\;\;\;\;\;\;\;\;\;\;\;\;;0<D<1$$ (8)

The notation D represents the duty cycle. For the inductor current, $I_{L}$ and capacitor voltage, $V_{c}$ to work in CCM, several conditions must be fulfilled. The design of the DC-DC boost converter’s specifications starts with the selection of the inductor capacity L as given by Eq. (9).


$$L=\frac{V_{i}\times \left(V_{o}-V_{i}\right)}{\Delta I_{L}\times f_{s}\times V_{o}}$$ (9)

The mathematical expression of the inductor ripple current $\Delta I_{L}$ is given as follows:


$$\Delta I_{L}=(0.2\;to\;0.4)\times I_{o\;max}\times \frac{V_{o}}{V_{i}}$$ (10)

Meanwhile, the minimum value of the output capacitor $C_{o\;min}$ is expressed as follows:


$$C_{o\;min}=\frac{I_{o\;max}\times D}{f_{s}\times \Delta V_{o}}$$ (11)

$$\Delta V_{o}=ESR\times \left(\frac{I_{o\;max}}{1-D}+\frac{\Delta I_{L}}{2}\right),\;ESR=1.5\Omega$$ (12)

The notation of $f_{s}$, $I_{o\;max}$, $\Delta V_{o}$ and ESR stands for DC-DC Boost converter’s frequency switching, maximum output current, output voltage ripple, and the amount of allowable output capacitor equivalent series resistance, respectively.

## 5. Three-phase voltage source inverter

**Fig 5:**
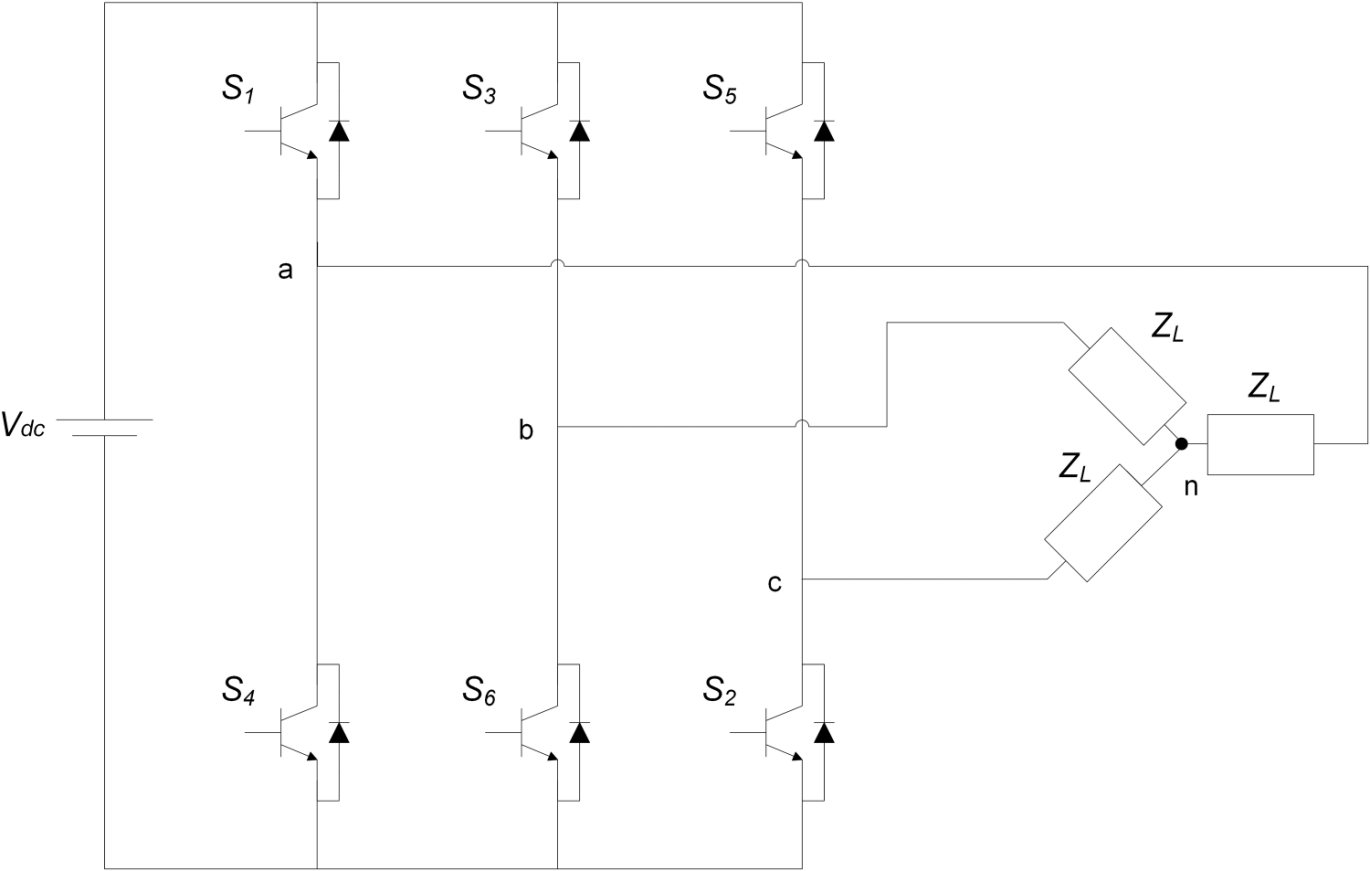
The schematic diagram of the three-phase VSI.

The conversion of an input DC voltage from a DC-DC boost converter into a symmetrical 3-phase sinusoidal AC waveform of the expected operating line frequency and voltage magnitude was achieved in this study using the 3-phase voltage source inverter (VSI) shown in Fig 5.

Two types of conduction modes can be applied to the three-phase VSI switches, these are the 120 ° and 180 °  conduction. The preferred method in this work is the 180 ° conduction mode because of its better switch utilization. For the three-phase VSI, the RMS line voltage $V_{Line}$ that corresponds to the DC-DC boost converter’s input DC voltage, $V_{dc}$ is given by Eq. (13) [31].


$$V_{Line}=m_{a}\frac{\sqrt{3}}{2\sqrt{2}}V_{dc}$$ (13)

where the modulation index is given as $m_{a}$. The expression of the instantaneous line-to-line voltage $V_{ab}$ in a Fourier series is given as:


$$V_{ab}=\sum_{n=1,3,5,\ldots }^{\infty }\frac{{4V}_{dc}}{n\pi }\sin\frac{n\pi }{3}\sin n\left(\omega t+\frac{\pi }{6}\right)$$ (14)

where the phase angle is given as ωt in radian while n represent the number of harmonics. Meanwhile, both $V_{bc}$ and $V_{ca}$ can be found in Eq. (14) by phase shifting $V_{ab}$ by 120^o^ and 240^o^, respectively. Therefore,


$$V_{bc}=\sum_{n=1,3,5,\ldots }^{\infty }\frac{{4V}_{dc}}{n\pi }\sin\frac{n\pi }{3}\sin n\left(\omega t-\frac{\pi }{2}\right)$$ (15)

$$V_{ca}=\sum_{n=1,3,5,\ldots }^{\infty }\frac{{4V}_{dc}}{n\pi }\sin\frac{n\pi }{3}\sin n\left(\omega t-\frac{7\pi }{6}\right)$$ (16)

Furthermore, the instantaneous phase voltages for a Y-connected load are given in Fourier series expressions as follows;


$$V_{an}=\sum_{n=1,3,5,\ldots }^{\infty }\frac{{4V}_{dc}}{\sqrt{3}n\pi }\sin\frac{n\pi }{3}\sin n(\omega t)$$ (17)

$$V_{bn}=\sum_{n=1,3,5,\ldots }^{\infty }\frac{{4V}_{dc}}{\sqrt{3}n\pi }\sin\frac{n\pi }{3}\sin n\left(\omega t-\frac{2\pi }{3}\right)\;$$ (18)

$$V_{cn}=\sum_{n=1,3,5,\ldots }^{\infty }\frac{{4V}_{dc}}{\sqrt{3}n\pi }\sin\frac{n\pi }{3}\sin n\left(\omega t-\frac{4\pi }{3}\right)$$ (19)

Meanwhile, Eq. (20) is the formula for both either voltage or current total harmonic distortion (THD) and is expressed as follows;


$$THD=\sqrt{\sum_{n=2}^{\infty }{\left(\frac{M_{n\;rms}}{M_{1\;rms}}\right)}^{2}}\times 100$$ (20)

where $M_{n}$ is the RMS value of the harmonic component of $n^{th}$ of the quantity M (voltage or current). On the other hand, the efficiency, η of the system can be calculated by using the following equation,


$$\eta =\frac{Output\;power,\;P_{o}}{Input\;power,\;\;P_{i}}\times 100\;(\;\%)$$ (21)

As mentioned previously, since the three-phase VSI is selected as the main inverter topology in this research, the most commonly coupled switching control technique is pulse-width modulation (PWM).

## 6. Space-vector pulse-width modulation

**Fig 6:**
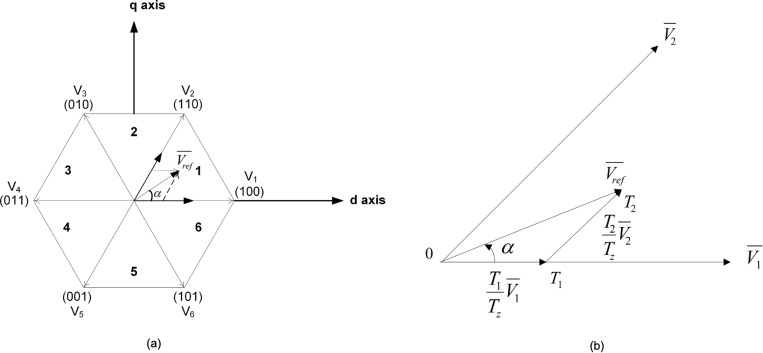
(a) Switching vectors and (b) Reference vector in Sector 1 of SVPWM.

In SVPWM, three-phase quantities (a,b,c) are represented as vectors in two-dimensional (d,q) reference frame as seen in Fig 6(a) and 6(b), respectively [32,33].

Adjacent vectors are used to calculate the reference voltage vector, $V_{ref}$ as seen in Fig 6(b). The calculation of the stationary dq reference frame is done using Eq. (22) [34].


[VdVq]=23⁢1-12-12032-32⁢VanVbnVcn (22)

The value of dq is used to determine the magnitude of the reference voltage vector $V_{ref}$ as given in Eq. (23).


$$\left|V_{ref}\right|=\sqrt{V_{d}^{2}+V_{q}^{2}}$$ (23)

Eq. (24) is used to calculate the angle between these two adjacent vectors.


$$\alpha =\tan^{-1}\left(\frac{V_{q}}{V_{d}}\right)=\omega t=2\pi ft$$ (24)

The required switching period at sector 1 is expressed thus:


$${\;T}_{z}\times V_{ref}=\left(T_{1}\times V_{1}+T_{2}\times V_{2}\right)$$ (25)

Hence, the switching time of SVPWM at any sector can be estimated using Eqs. (26)–(28).


$$T_{1}=\frac{\sqrt{3}\times T_{z}\times \left|V_{ref}\right|}{V_{dc}}\left(\sin\frac{n}{3}\pi \cos\alpha -\cos\frac{n}{3}\pi \sin\alpha \right)$$ (26)

$$T_{2}=\frac{\sqrt{3}\times T_{z}\times \left|V_{ref}\right|}{V_{dc}}\left(-\cos\alpha \times \sin\frac{n-1}{3}\pi +\sin\alpha \times \cos\frac{n-1}{3}\pi \right)$$ (27)

$$T_{0}=T_{Z}-T_{1}-T_{2}$$ (28)

## 7. Interfacing line filters parameters design

**Fig 7:**
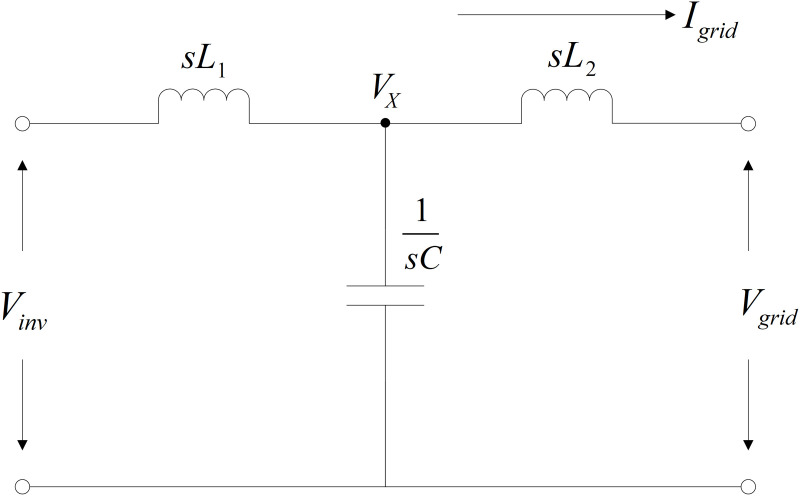
The equivalent circuit of LCL filters in s-domain.

In the GPV generation system, the presence of power electronic components such as power Mosfet or IGBT which are modulated by the high-frequency PWM. These high-frequency PWM modulations resulted in a high-rate change of voltage and current over time. The voltage and current generated from the inverter especially consist of high harmonic order and if it flows into the power grid, will create harmonic pollution. For this reason, line filters need to be installed. According to various research works as discussed in [35–37], among several passive filter configurations, LCL filters have the best high-frequency attenuation characteristics as compared to L and LC filters. Therefore, the LCL filter configuration is applied to the proposed two-stage three-phase GPV generation system. The design of three-phase LCL power filters begin with the mathematical derivation to obtain the transfer function of filters. The three-phase LCL filter configuration is re-drawn in s-domain circuitry as illustrated in Fig 7.

Here, $V_{inv}\;$ denoted as the inverter voltage, $sL_{1}$ represents inductor value on the inverter side, $sL_{2}\;$ represents the inductor value on the grid side, 1/sC  is the total capacitance, and $V_{grid}\;$ is symbolized as the grid voltage. By applying Kirchhoff’s current law at point X, the mathematical expression is given as follows:


$$\frac{V_{inv}-V_{X}}{sL_{f1}}=I_{grid}+V_{X}sC_{f}$$ (29)

The voltage expression at X junction in terms of grid current, $I_{grid}$ is given by the following equation;


$$V_{X}=I_{grid}sL_{f2}$$ (30)

Solving both Eqs. (29) and (30) simultaneously will result in the arrangement of the transfer functions equation as follows:


$$\frac{I_{grid}}{V_{inv}}=\frac{1}{s^{3}L_{f1}L_{f2}C_{f}+s\left(L_{f1}+L_{f2}\right)}$$ (31)

Lets,


$$L_{f1}+L_{f2}=L_{f}$$ (32)

and


$$\frac{L_{f1}L_{f2}}{L_{f1}+L_{f2}}=L_{R}$$ (33)

Again, solving Eq. (31) until Eq. (33) respectively brings out the new mathematical expression as shown in Eq. (34).


$$\frac{I_{grid}}{V_{inv}}=\frac{1}{sL_{f}\left(s^{2}L_{R}C+1\right)}$$ (34)

The mathematical expression of the resonant frequency $\omega_{R}$ is given as follows:


$$\omega_{R}=\frac{1}{\sqrt{CL_{R}}}$$ (35)

The selection of the switching frequency, $f_{s}$ is based on several factors, such as component constraints, size, cost of the components, and thermal considerations [38]. The switching frequency for the three-phase VSI was selected as  10kHz. As a rule of thumb, the resonant frequency, $f_{r}$ is defined as follows:


$$f_{r}=\frac{f_{s}}{10}$$ (36)

Therefore,


$$f_{r}=1000Hz$$

Based on the IEEE grid code standards which state that the total reactive power, $Q_{T}$ should be less than 5% of the system’s rated power,  S, therefore:


$$Q_{T}=\frac{V_{P}^{2}}{\frac{1}{2\times \pi \times f_{n}\times C_{f}}}$$ (37)

$$\frac{5}{100}\times S=\frac{V_{P}^{2}}{\frac{1}{2\times \pi \times f_{n}\times C}}$$

The designing of the LCL filter is continued by determining the value of line filter inductance, $L_{f}$. Referring to Eq. (34) and given that the notation $\;s=j\omega_{sw}$,


$$\frac{I_{grid\;(sw)}}{V_{inv}}=\frac{1}{j\omega_{sw}L_{f}\left(1+\frac{{\left(j\omega_{sw}\right)}^{2}}{\omega_{r}^{2}}\right)}$$ (38)

By mathematical definition, $j^{2}=-1$ and by taking the magnitude, Eq. (38) becomes:


$$\left|\frac{I_{grid\;(sw)}}{V_{inv\;(sw)}}\right|=\left|\frac{1}{j\omega_{sw}L_{f}\left(1+\frac{{\left(j\omega_{sw}\right)}^{2}}{\omega_{r}^{2}}\right)}\right|$$ (39)

Rearranging Eq. (39) gives the magnitude of inductance, $L_{f}\;$as follows:


$$\left|L_{f}\right|=\left|\frac{1}{\omega_{sw}\frac{I_{grid\;(sw)}}{V_{inv\;(sw)}}\left(1-\frac{\omega_{sw}^{2}}{\omega_{r}^{2}}\right)}\right|$$ (40)

By considering all the requirements set by the established grid code standards in the whole calculation design for line LCL passive filters, therefore, the single conductor value, $L_{f}$ is fixed at 500μH and the value for single capacitance, $C_{f}$ is set at 100.28μF. These values will be used for the interfacing passive power filters in this research work.

## 8. Grid synchronization

Two control loops—an exterior voltage control loop and an internal current control loop—are used in the design of the three-phase GPV generation system’s control mechanism. The regulation of the injected current from the inverter to the grid is performed by the current control loop; this loop also keeps the injected current in phase with the grid voltage to ensure the achievement of the unity power factor. The voltage control loop is used to regulate the output power from PV modules to the grid; it also balances the power flow. A crucial part of grid synchronization is the phase-locked loop (PLL) control mechanism, which is used in the synchronous reference frame (SRF). The main advantages of SRF are that the fundamental components of three-phase waveform signals (abc) are converted into DC signals  (dq), consequently reducing the computational difficulty. The phase angle, θ and rotation frequency, ω are the two critical pieces of information extracted from the grid voltages by using the SRF-PLL method. This three-phase abc natural frame is converted into three constant DC components by using a dq transformation which is also known as synchronous reference frame or SRF in short form. The three DC components in the SRF plane are denoted as direct  (d), quadrature (q) and zero  (0), respectively. The relationship between these two frames in terms of voltages and currents is given by the following expression:


$$\left[\begin{array}{c} V_{d}\\ V_{q}\\ V_{0} \end{array}]=[T][\begin{array}{c} V_{a}\\ V_{b}\\ V_{c} \end{array}\right]$$ (41)

$$\left[\begin{array}{c} I_{d}\\ I_{q}\\ I_{0} \end{array}]=[T][\begin{array}{c} I_{a}\\ I_{b}\\ I_{c} \end{array}\right]$$ (42)

$$\left[T]=\sqrt{\frac{2}{3}}[\begin{array}{ccc} \sin\alpha & \sin\left(\alpha -\frac{2\pi }{3}\right) & \sin\left(\alpha +\frac{2\pi }{3}\right)\\ \cos\alpha & \cos\left(\alpha -\frac{2\pi }{3}\right) & \cos\left(\alpha +\frac{2\pi }{3}\right)\\ \frac{1}{\sqrt{2}} & \frac{1}{\sqrt{2}} & \frac{1}{\sqrt{2}} \end{array}\right]$$ (43)

The matrix [T] is sometimes called Park Transformation. The active, P and reactive powers, Q injected by the three-phase VSI inverter can be calculated in dq frame by using the following expression:


$$P=V_{d}I_{d}+V_{q}I_{q}$$ (44)

$$Q=V_{d}I_{q}+V_{q}I_{d}$$ (45)

In SRF, by using a PLL technique, the grid frequency is locked in such a way that the quadrature component, (q) is set to zero, i.e., $V_{q}=0$. Therefore, the real and reactive power expressions can be simplified to:


$$P=\frac{3}{2}V_{d}I_{d}$$ (46)

$$Q=\frac{3}{2}V_{d}I_{q}$$ (47)

From the above expression, since $V_{d}$ is kept constant, the real power, P injection into the grid can be accomplished by regulating the value of DC-Link voltage through the control of direct axis current$,\;I_{d}$. On the other hand, the reactive power, Q depends on the value of ${\;\;I}_{q}$. Based on the principle of power balance between input and output power, the voltage dynamics of DC-Link capacitor is given by:


$$\frac{d}{dt}V_{dc}^{2}=\frac{2}{C}\left(P_{i}-P_{o}\right)$$ (48)

The reference direct axis current $I_{d}^{*}$ is extracted from the error difference between $P_{i}$ and${\;\;P}_{o}$ using a proportional-integral (PI) controller Integrating Eq. (48) with respect to time gives the new mathematical expression depicted as:


$$I_{d}^{*}=\;\;\;K_{p}\left(V_{dc}^{*}-V_{dc}\right)+K_{I}\int \left(V_{dc}^{*}-V_{dc}\right)dt$$ (49)

where $I_{d}^{*}$ is designated as. The voltage expressions in dq−SRF can be translated as follows;


$$V_{d}^{*}=K_{p}\left(I_{d}^{*}-I_{d}\right)+K_{i}\int \left(I_{d}^{*}-I_{d}\right)dt-\omega LI_{q}+V_{d}$$ (50)

$$V_{q}^{*}=K_{p}\left(I_{q}^{*}-I_{q}\right)+K_{i}\int \left(I_{q}^{*}-I_{q}\right)dt+\omega LI_{d}+V_{q}$$ (51)

where $V_{d}^{*}$, $V_{q}^{*}$, $I_{d}^{*}$ and $I_{q}^{*}$ are grid voltage and grid current’s DC components, respectively; $V_{d}$, $V_{q}$, $I_{d}$ and $I_{q}$ are the inverter output waveforms’ DC components for achieving unity power factor; it is required that the q-axis quadrature current component is equal to zero consequently the reference command reactive current $I_{q}^{*}$ is set to zero $\left(I_{q}^{*}=0\right)$ [39].

## 9. Cascaded delay signal cancellation

**Fig 8:**
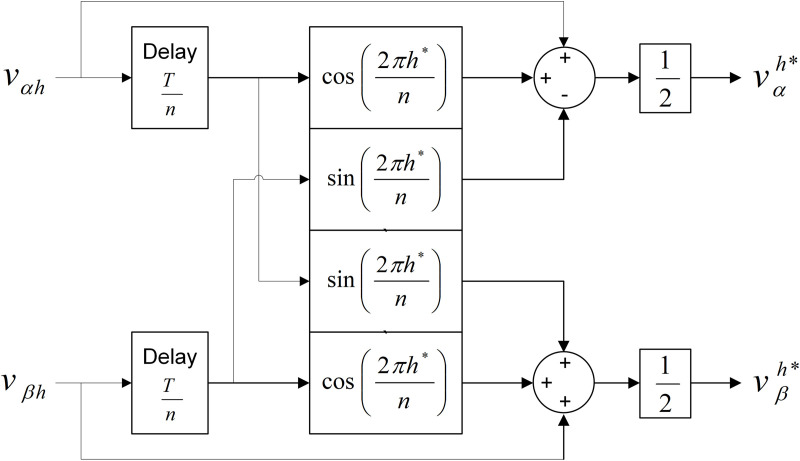
Details of DSC block diagram configuration.

The introduction of cascaded delay signal cancellation or CDSC is based on the statement that the PLL is unable to perform accurately during the conditions where the line grid voltage is highly distorted [40]. The CDSC operator in dq reference frame introduces the time delay expression into the SRF-PLL configurations’ control loop. The PLL dynamic performance is adversely affected by the introduction of this in-loop delay. Therefore, the stability of SRF-PLL is ensured by relocating the equivalent of CDSC operators into αβ reference frames using these equations [41];


$$\left[\begin{array}{c} {DSC}_{n}\left[v_{\alpha h}\right]\\ {DSC}_{n}\left[v_{\beta h}\right] \end{array}]=[\begin{array}{cc} \cos h*\theta & -\sin h*\theta \\ \sin h*\theta & \cos h*\theta \end{array}]*[\begin{array}{c} {DSC}_{n}\left[v_{dh}\right]\\ {DSC}_{n}\left[v_{qh}\right] \end{array}\right]$$ (52)

$${DSC}_{n}\left[v_{dqh}\right]=\frac{1}{2}\left[v_{dq}h(\omega t)+v_{dq}h-\frac{T}{n}\right]$$ (53)

where ω and h are the fundamental angular frequency and the harmonic order respectively; $h^{*}=\pm h$ is the positive sequence component of the harmonic frequency that the CDSC operator needs to extract; ‘${+}^{\prime }and$ ‘${-}^{\prime }$ represent positive and negative sequence signals respectively, and θ=ωt. The equations are then translated into the block diagram configuration as shown in Fig 8.

## 10. Specifications of the proposed three-phase GPV generation system

**Fig 9:**
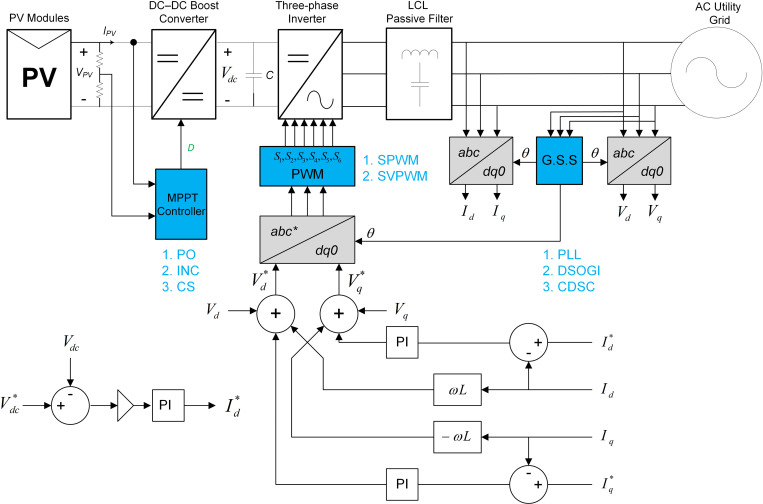
The overall block diagram of the proposed GPV generation system.

As mentioned earlier, the key objective of the research work is to design and model a two-stage three-phase GPV generation system that can deliver the real power of 100 kW from the PV system to the utility grid under various atmospherics and grid fault conditions. The overall block diagram of the proposed GPV generation system is illustrated in Fig 9.

The generated output voltage and injected current waveforms from the PV system need to comply with the grid code standards and regulations. The list of design parameters for the proposed two-stage three-phase GPV generation system was tabulated in Table 2.

**Table 2:** The overall list of the parameters for the proposed three-phase GPV System.

Section	Parameter	Value
**PV Modules**	Reference Model	1Soltech 1STH-215-P
	Nominal peak power, $P_{\max}$	213.15W
	The voltage at maximum power, $V_{mp}$	29V
	Current at maximum power, $I_{mp}$	7.35A
	Short-circuit current, $I_{sc}$	7.84A
	Open-circuit voltage, $V_{oc}$	36.3V
	Number of modules in series, $n_{s}$	10
	Number of modules in parallel, $n_{p}$	47
**DC-DC boost converter**	Rated Power, P	100kW
	Input voltage, $V_{i}$	$250V_{DC}-350V_{DC}$
	Boost Inductor, $L_{b}$	1.45mH
	Switching frequency, $f_{s}$	5kHz
	Boost Input Capacitor, $C_{i}$	220uF
	Boost Output Capacitor, $C_{b}$	3227uF
	Output voltage, $V_{dc}$	$600V_{DC}$
**Three-phase VSI**	Modulation Index, $m_{a}$	1
	Switching frequency, $f_{s}$	10kHz
	PWM Technique	SPWM
	Output Phase Voltage, $V_{o\;rms}$	${230V}_{LN}$
**Line Filters**	Line Inductance, $L_{f}$	500uH
	Line Capacitance, $C_{f}$	100.28uF
**Three-phase utility grid**	Power Rating	10000VA
	Operating Line Voltage, $V_{grid}$	${400V}_{LL}$
	Operating line frequency, f	50Hz

## 11. Results and discussion

**Fig 10:**
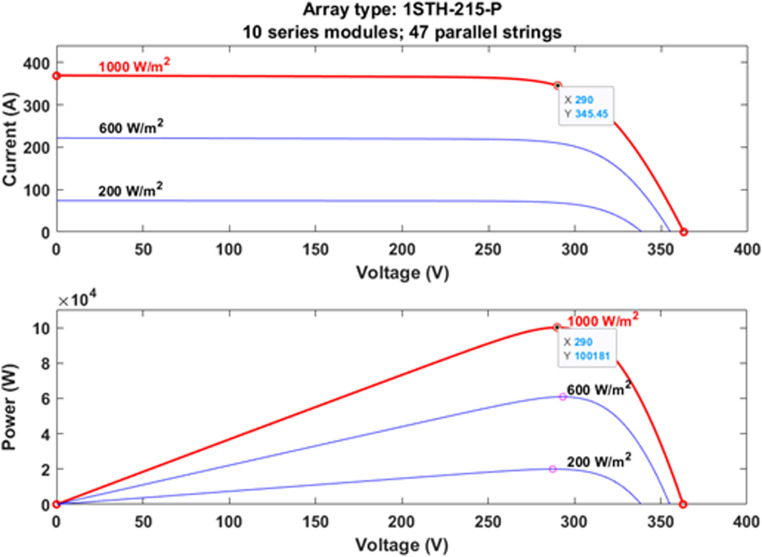
The PV characteristics of I−V and the P−V at various irradiation levels.

The PV module from 1Soltech (1STH-215-P) is selected as the reference PV model in this research. Based on the datasheet, the voltage value at the maximum power point of a 1Soltech (1STH-215-P) PV module, $V_{mpp}$ is  29 V. Meanwhile, the PV module current at the maximum power point, $I_{mpp\;}$ is rated at  7.35 A. Therefore, the maximum power rating, $P_{mpp}$ for this single PV module is  213.15W. In this research, the surface temperature, T is set at a constant value of $25^{\circ }\text{C}$ since it does not have much effect on the generation of PV current, and its analyses are not covered in this research work. The designated PV modules are poised to generate 100 kW of real power, P. Therefore, it requires PV modules to be connected in 47 parallel strings and 10 pieces of PV modules in series connections to form PV arrays. The PV arrays are tested at three different levels of irradiation, G which are fixed at 1000 $W/m^{2}$, 600 $W/m^{2}$ and 200 $\;W/m^{2},$ respectively. Fig 10 shows the relationship between I−V and P−V characteristics of PV arrays according to the selected value of irradiation level.

**Fig 11:**
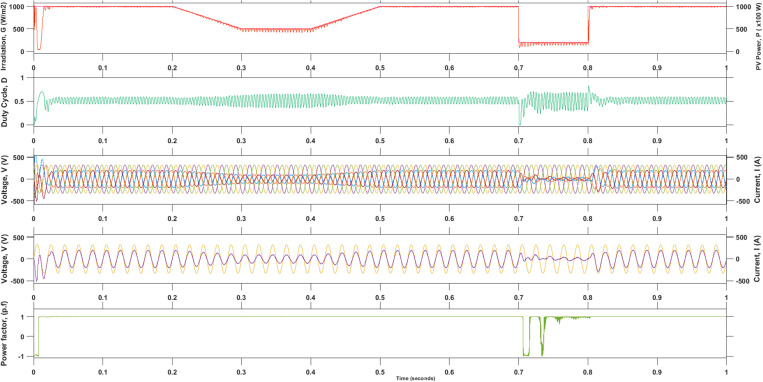
The overall system’s response to the variation of irradiation levels.

The overall system performance during abrupt atmospheric changes is presented in Fig 11. The most upper waveform in red color indicates the irradiation value and its corresponding output PV power generated. The next row (green color) indicates the motion from the duty cycle, D  response due to the changes in input parameters produced by the maximum power point tracking technique (MPPT) algorithm. The following rows indicate the equivalent voltage and the injected current waveforms of the proposed GPV system. The subsequent row displays the phase R voltage and the component of the injected current waveform is in-phase even though in case of irradiation changing. The last row presents the value of the power factor, PF. The PF is kept constant at a value of 1.0 except during irradiation drops occurred at t=0.7s until t=0.8s  as it is unable to provide good PF reading due to the very low generation and distorted current waveform.

**Fig 12:**
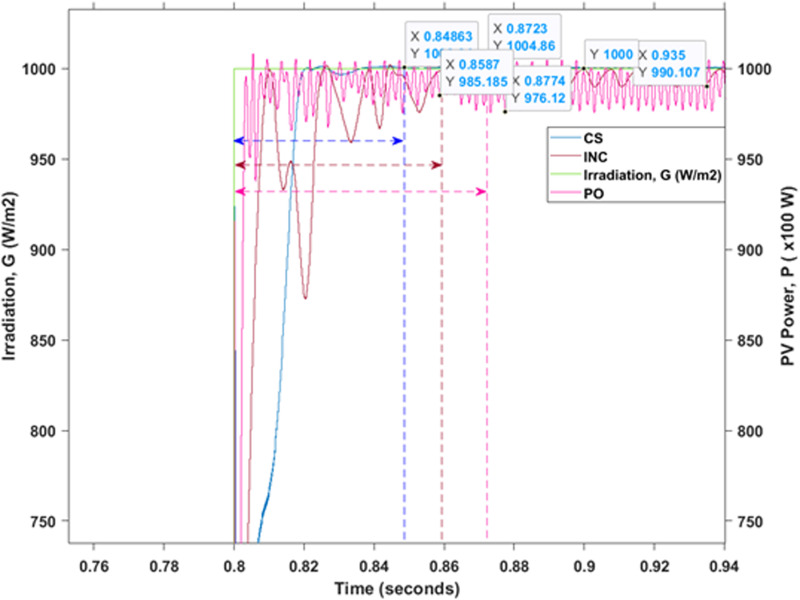
Performance for the generated output PV power of all three studied MPPT methods.

Detailed performance comparison of the tracking response and the magnitude of PV power oscillation of all the selected MPPT methods are further analysed as illustrated in Fig 12. Based on the result provided, the highest magnitude of PV power oscillation that occurred in generated output PV power is produced by the PO MPPT method. The difference between the two points of power oscillation is almost 3000 W which is a huge loss in terms of power efficiency. As compared to both of its counterparts, the CS MPPT technique offers huge advantages for PV maximum power point extraction, especially with the high degree of tracking efficiency and almost unnoticeable effect on duty cycle variation in the event of sudden changes of irradiation that took place between the period of t=0.7s until t=0.8s. Furthermore, during the transient atmospheric condition (sudden drops of irradiation level), the CS MPPT method is the fastest in terms of speed to reach the steady-state form (t=0.848 s) which is one of the important features of improving the maximum PV power tracking efficiency. In addition, the CS produces very small (approximate ±1W) PV power oscillation that consequently resulted in improved overall system efficiency. Moreover, the CS created less if not zero amplitude variation of D even in the event of absurd atmospheric situations and was able to reduce the electrical stress on the power switches. Therefore, the CS algorithm MPPT technique produces the best result in terms of power oscillation, tracking efficiency, and ability to track MPP even under the most absurd atmospheric conditions.

**Fig 13:**
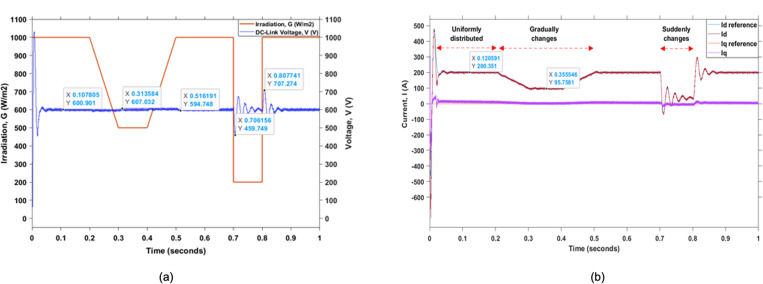
(a) Response of DC-link voltage and (b) Current controller during variation of irradiation.

The DC-link voltage, $V_{dc-link}$ should be constant in case of any input fluctuations. As illustrated in Fig 13(a), in the beginning, the level of solar irradiation is poised at a steady level and starts to gradually decrease at t=0.2 s and reached an irradiation level of 500 W/m2  at t=0.3 s. In the event of absurd atmospheric conditions which occurred at t=0.7s until t=0.8s, the $V_{dc-link}$ waveform was still able to be kept at a constant value of 600 V even though a noticeable fluctuation occurred due to the controller action which required a certain time to achieve a steady-state voltage level. In contrast, there is no current oscillation that would be noticed during gradual changes of irradiation that took place at t=0.2 s until t=0.5 s  as demonstrated in Fig 13(b). However, at the time, t=0.7 s  until t=0.8 s, certain transient was been identified for the direct current component, $I_{d}$; it occurred during sudden changes in irradiations. On the other hand, the value of the quadrature current component, $I_{q}$ is kept at nearly zero values.

**Fig 14:**
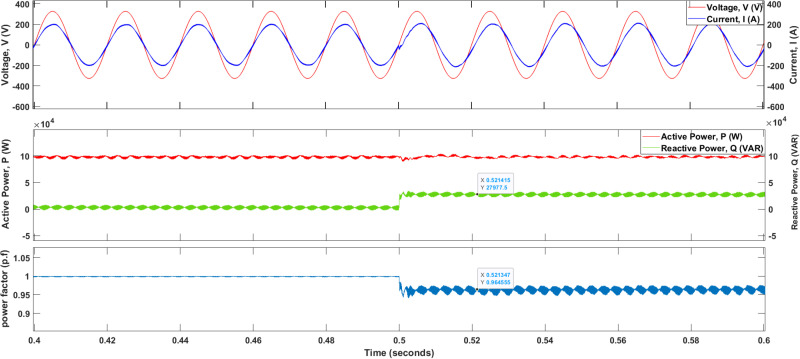
Delivery of reactive power, Q to the grid after time, t=0.5s.

Moreover, as displayed in Fig 14, the maximum amplitude of the grid voltage is 325 V whereas the maximum amplitude of the injected current waveform showed a reading of   200 A. Furthermore, it can be observed that the waveform of the injected current (phase R) is in phase with the waveform of grid voltage, $V_{grid}$ and as a consequence, it kept the power factor (PF) near unity or  1. Furthermore, the reactive power, Q can also be delivered (28kVAR) to the grid by controlling the value of the quadrature current, $I_{q}$. It can be noticed that there is a drop in the power factor value (p.f=0.96) after time t=0.5 s .

**Fig 15:**
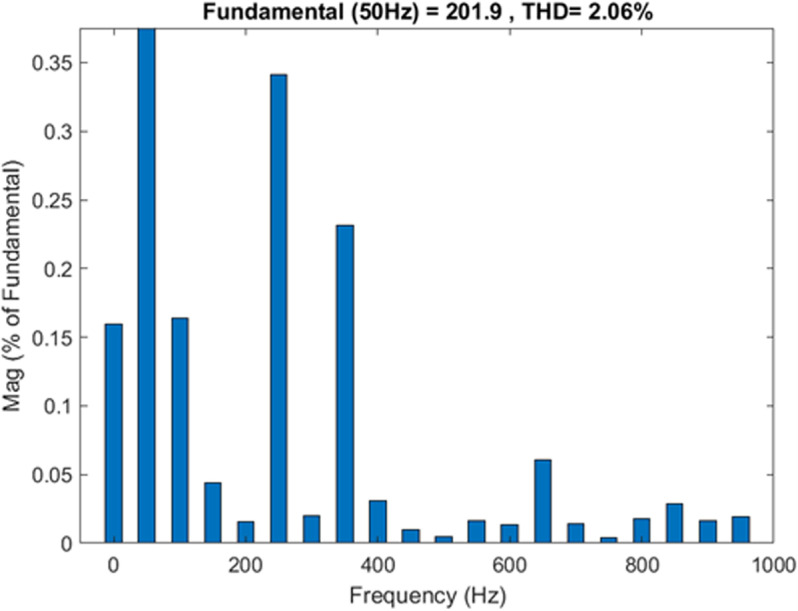
The ${THD}_{i}$ value for the injected current waveforms using SVPWM.

The THD level for the injected current waveform using SVPWM switching as displayed in Fig 15, is around 2.06 %; this value is below the grid code limit of 5 %. The recorded amplitude of the current waveform at the fundamental frequency is 200.20 A.

**Fig 16:**
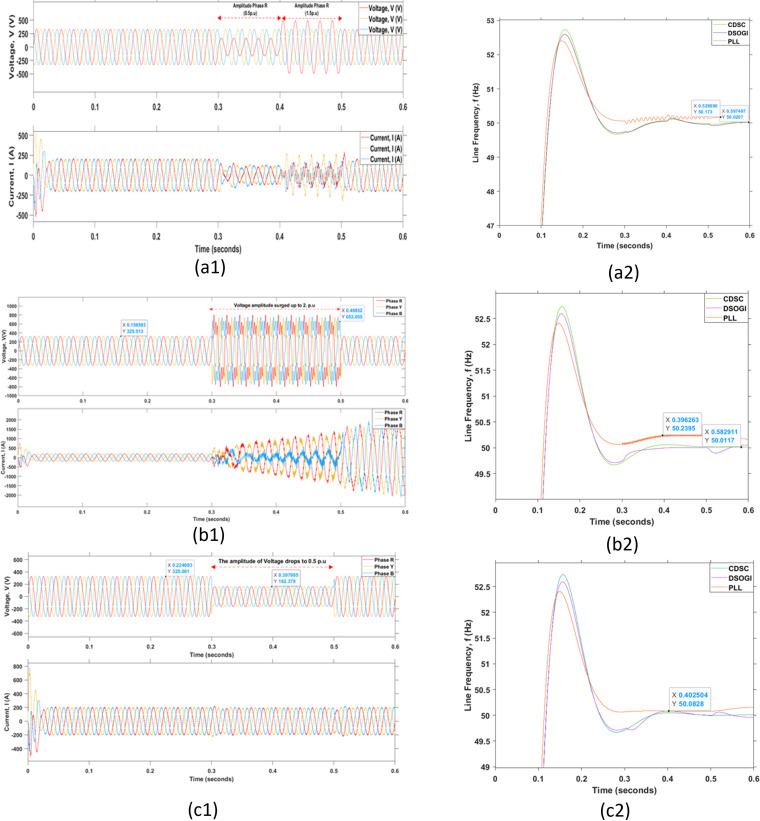
Grid disturbances due to voltage variations. (a1) Voltage unbalance, (b1) Voltage surge, and (c1) Voltage dip with their corresponding outcomes shown in (a2), (b2), and (c2) respectively.

**Fig 17:**
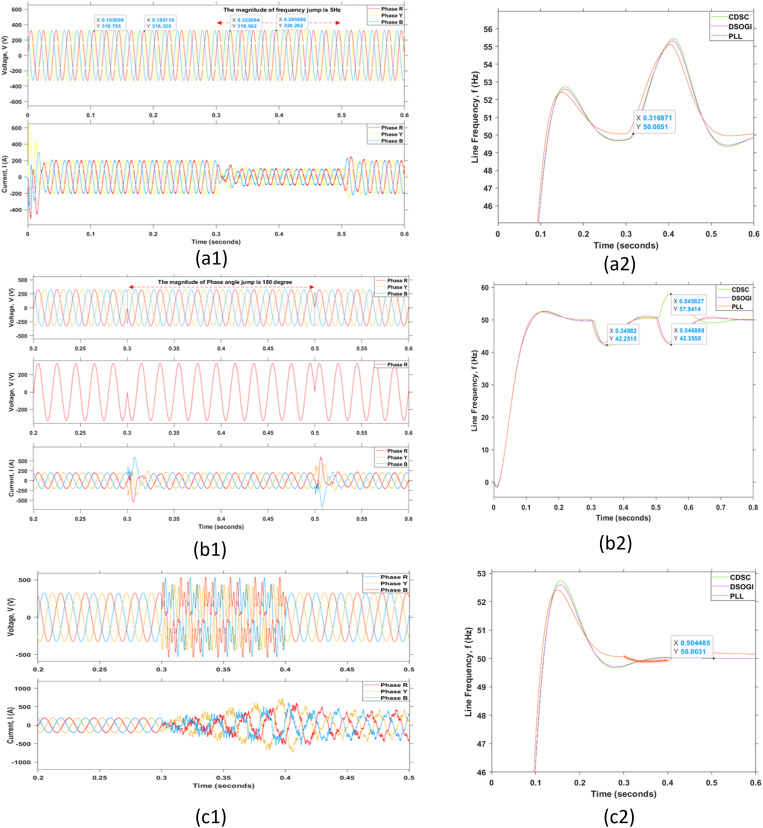
Grid disturbances due to frequency, phase angle jump, and harmonic distortion. (a1) Frequency jump, (b1) Phase angle jump, and (c1) Harmonic distortion with their corresponding outcomes shown in (a2), (b2), and (c2) respectively.

**Fig 18:**
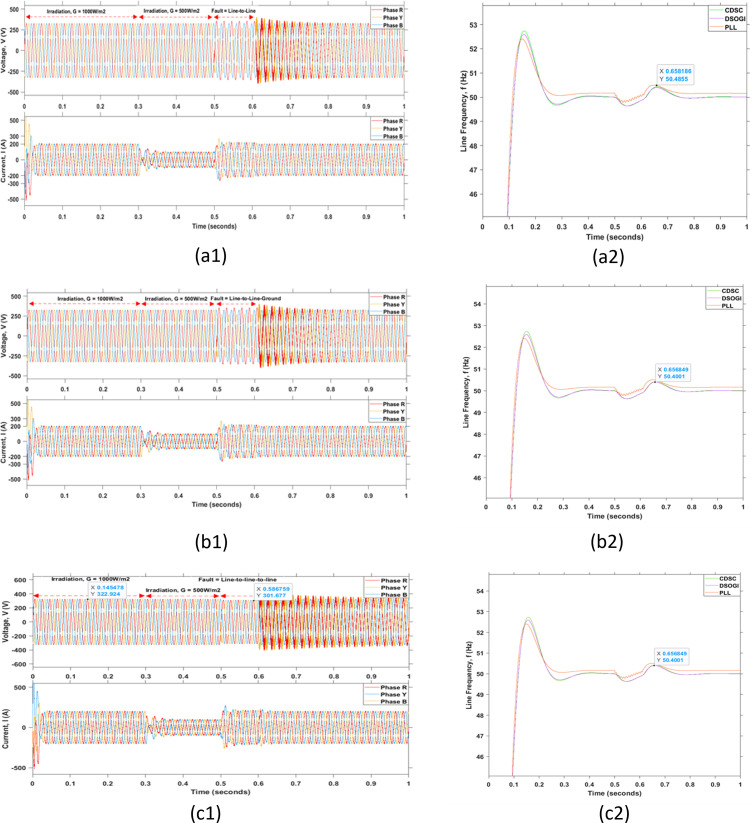
Grid disturbances due to various line faults. (a1) Line-to-line fault, (b1) Line-to-line-to-ground fault, and (c1) Line-to-line-to-line fault with their corresponding outcomes shown in (a2), (b2), and (c2) respectively.

The effectiveness of the grid synchronization mechanism during fault conditions is vital. The grid fault is defined as the grid in a faulty state when the peaks of voltage or line frequency are in severe states and exceed the maximum margins mentioned in the standards. The grid fault or grid disturbance can be categorized into several types of faults such as the grid fault due to the voltage unbalance, voltage surge, voltage dip, frequency jump, phase angle jump, harmonic distortions, and grid-lines faults as illustrated in Figs 16–18, respectively.

**Fig 19:**
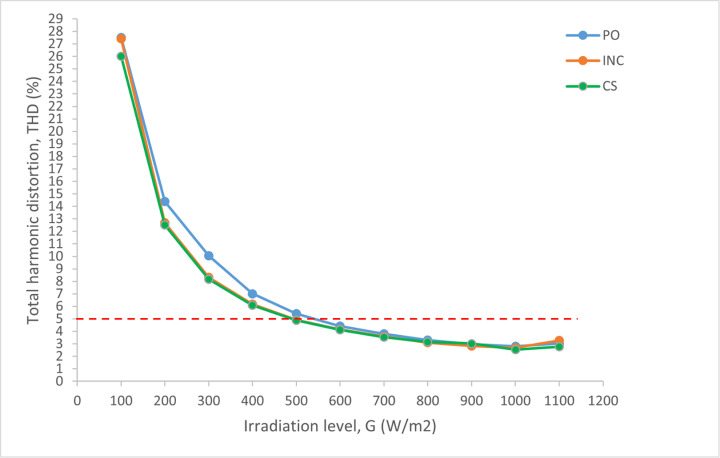
Comparison of ${THD}_{i}$ between all selected MPPT methods.

The performances of all the studied MPPT algorithms are analyzed in terms of ${THD}_{i}\;$ generation. In Fig 19, it is proved that the CS is able to improve the ${THD}_{i}\;$ and reach the recommended level at the irradiation level of 500 W/m2.

**Fig 20:**
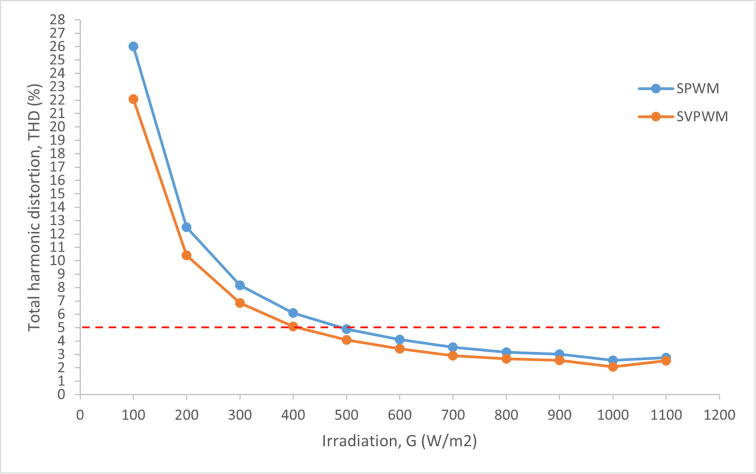
Comparison of ${THD}_{i}$ between SPWM and SVPWM switching topologies.

Moreover, as shown in Fig 20, the SVPWM switching topology reached the acceptable ${THD}_{i}$ limit of less than 5 % of the rated inverter input current at the irradiation level of 400 W/m2 as compared to the SPWM which touched the 5 % ${THD}_{i}$ limit at the irradiation level of 500 W/m2. Hence, with the utilization of the SVPWM, the proposed GPV generation system is able to deliver an additional good quality of power from the PV system to the utility grid even at a low irradiation level. On top of that, the SVPWM proved to be more superior and produced a low value of ${THD}_{i}$ at every irradiation level as compared to the SPWM switching technique.

A detailed comparison of the individual performance between the proposed GPV generation system and similar research works is tabulated in Table 3. The proposed 100 kW two-stage three-phase GPV generation system comprises controllers of the Cuckoo Search (CS) MPPT technique; the SVPWM switching topology in combination with the CDSC synchronization mechanism is the most effective PV-grid system configuration as it proved to have better MPPT tracking efficiency of 96 % and requires only 0.05 s to reach steady-state in the course of transient, ${THD}_{i}$ level of 2.06 % and the proposed system is able to work efficiently even in the case of grid fault conditions.

**Table 3:** A performance validation of a proposed system with similar research work.

Parameters	[42]	[43]	[28]	[44]	[45]	Proposed System
System Rating	10kW	250kW	8kW	10kW	15kW	100kW
MPPT	NA	INC	P&O	P&O	P&O	CS
DC-DC Converter	NA	NA	DC-DC Boost	DC-DC Boost	DC-DC Boost	DC-DC Boost
Three-phase Inverter	Single Stage	Single Stage	Two Stage	Two Stage	Two Stage	Two Stage
Switching Topologies	SVPWM	SPWM	SPWM	SVPWM	SPWM	SVPWM
Synchronization Mechanism	PLL	MFC	SPLL	PLL	DSOGI	CDSC
Atmospherics profile	NA	Absurd condition	Steady-state	Absurd condition	Steady-state	Absurd condition
Grid Condition	Grid fault condition	Steady-state grid condition	Steady-state grid condition	Steady-state grid condition	Grid fault condition	Grid fault condition
Efficiency of MPPT, η	NA	95%	95%	93%	NA	96%
Time requires to reach steady-state	0.1s	0.05s	0.5s	0.65s	NA	0.05s
Oscillation	PV power oscillation occurred	PV power oscillation occurred	PV power oscillation occurred	PV power oscillation occurred	PV power oscillation occurred	No PV power oscillation occurred
${THD}_{i}$	4.33%	2.48%	NA	4.89%	3.2%	2.06%

## 12. Conclusion

The two-stage three-phase GPV generation system with a power rating of 100 kW has been successfully designed, modeled, and analyzed in this research work. To attain the maximum 100 kW  active power delivery from the PV arrays into the utility grid, the proposed GPV generation system is constructed based on the two-stage power circuitry topology. The two-stage power circuitry topology is interpreted as having a stage-by-stage power conversion from DC to AC forms. Based on the results, the designated two-stage three-phase GPV generation system was able to deliver a total of 100181 W from the PV system to the utility grid at the irradiation level of $1000\;W/m^{2}$. Furthermore, this circuitry configuration reduced the controller complexity where each of the individual local controllers (MPPT, DC-DC Boost, Inverter, Grid synchronization) was able to perform the designated operation effectively. As a consequence, it optimized the overall reliability of the proposed GPV generation system. Moreover, with the suitable controller parameters, the ${THD}_{i}$ for the injected current waveforms at the irradiation level of 1000W/m2 is measured with an average value of around 2 % of allowable $\;{THD}_{i}$, thus complied with the grid code standards. Furthermore, it is proved that a designated 100 kW two-stage three-phase GPV generation system is combined with improved local controllers which are made up of an advanced CS MPPT, SVPWM as well as the CDSC grid synchronization mechanism is the most effective control mechanism against the absurd atmospherics and various grid fault conditions.
